# Functional Roles of the Interaction of APP and Lipoprotein Receptors

**DOI:** 10.3389/fnmol.2017.00054

**Published:** 2017-03-01

**Authors:** Theresa Pohlkamp, Catherine R. Wasser, Joachim Herz

**Affiliations:** ^1^Department of Molecular Genetics, UT Southwestern Medical CenterDallas, TX, USA; ^2^Center for Translational Neurodegeneration Research, UT Southwestern Medical CenterDallas, TX, USA; ^3^Department of Neuroscience, UT Southwestern Medical CenterDallas, TX, USA; ^4^Department of Neurology and Neurotherapeutics, UT Southwestern Medical CenterDallas, TX, USA

**Keywords:** LRP, APOE, LDL receptor gene family, neuromuscular junction, synapse, glutamate receptors, trafficking, amyloid beta

## Abstract

The biological fates of the key initiator of Alzheimer’s disease (AD), the amyloid precursor protein (APP), and a family of lipoprotein receptors, the low-density lipoprotein (LDL) receptor-related proteins (LRPs) and their molecular roles in the neurodegenerative disease process are inseparably interwoven. Not only does APP bind tightly to the extracellular domains (ECDs) of several members of the LRP group, their intracellular portions are also connected through scaffolds like the one established by FE65 proteins and through interactions with adaptor proteins such as X11/Mint and Dab1. Moreover, the ECDs of APP and LRPs share common ligands, most notably Reelin, a regulator of neuronal migration during embryonic development and modulator of synaptic transmission in the adult brain, and Agrin, another signaling protein which is essential for the formation and maintenance of the neuromuscular junction (NMJ) and which likely also has critical, though at this time less well defined, roles for the regulation of central synapses. Furthermore, the major independent risk factors for AD, Apolipoprotein (Apo) E and ApoJ/Clusterin, are lipoprotein ligands for LRPs. Receptors and ligands mutually influence their intracellular trafficking and thereby the functions and abilities of neurons and the blood-brain-barrier to turn over and remove the pathological product of APP, the amyloid-β peptide. This article will review and summarize the molecular mechanisms that are shared by APP and LRPs and discuss their relative contributions to AD.

## Lipoprotein Receptors

### Structure and General Physiological Properties

Besides the important role in lipid metabolism, members of the low-density lipoprotein (LDL) receptor family take part in a broad range of pre- and post-developmental functions in brain and play key roles in the pathogenesis of Alzheimer’s disease (AD). Much like the amyloid precursor protein (APP), members of the LDL receptor family are type-I membrane receptors with single-pass transmembrane (TM) domains that can be endocytosed, proteolytically processed and participate in a variety of protein interactions both inside and outside of the cell, including direct interactions with APP (May et al., 2005; Dieckmann et al., 2010). Lipoprotein receptors are involved in various mechanisms of APP-processing and Aβ-clearance in several cell types including neurons, astrocytes, endothelial cells of the blood brain barrier (BBB), and ependymal cells of the blood cerebrospinal fluid (CSF) barrier (BCSFB; reviewed by Hoe and Rebeck, 2008; Marzolo and Bu, 2009; Wagner and Pietrzik, 2012; Lane-Donovan et al., 2014).

**Figure 1:**
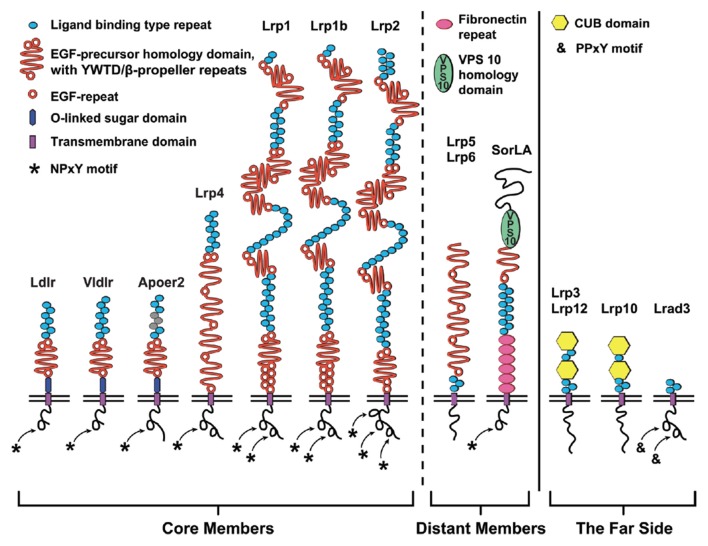
**The low-density lipoprotein (LDL) receptor family.** Schematic diagram depicting the domain structure of the LDL receptor family members classified as (*from left to right*): core, distant and the far side. The seven core members (*left*) are LDL receptor (Ldlr), very-LDL receptor (Vldlr), Apolipoprotein E (ApoE) receptor 2 (Apoer2/Lrp8), LDL receptor related protein (Lrp)-4 (Lrp4), Lrp1, Lrp1b and Lrp2. These members are classified as core members by the presence of at least one NPxY-motif (asterisk) and a combination of two classical LDL receptor domains: (1) N-terminal ligand binding domain composed of cysteine-rich ligand binding-type repeats (blue); and (2) epidermal growth factor (EGF)-precursor homology domain (orange) composed of EGF-repeats and YWTD/β-propeller domain. Ldlr, Vldlr and Apoer2 express an additional extracellular O-linked sugar (OLS) domain adjacent to the transmembrane (TM) segment. The more distant members (*middle*) are the NPxY-lacking Lrp5/Lrp6 and hybrid SorLA with additional Fibronectin repeats (pink) and importantly the VPS10p-sorting motif (green). Four very distant “far side” proteins (*right*, Lrp3, Lrp10, Lrp12, and Lrad3) only encode ligand binding-type repeats. Lrp3, Lrp10 and Lrp12 also contain atypical CUB-domain (binds Complement, Uegf and Bmp1). In addition to the OLS domains of Apoer2 and Vldlr, alternative splicing of Apoer2 produces splice variants lacking N-terminal ligand binding type repeats (repeats 4–6; Brandes et al., 2001; gray).

In the peripheral and central nervous system, lipoprotein receptors and APP interact to control developmental processes and synaptic function. These lipoprotein receptors are highly conserved—at least as far back in evolution as *C. elegans* (Yochem and Greenwald, 1993)—and are related by both structure and function (Krieger and Herz, 1994; Figure 1). The seven core members of this receptor family are the LDL receptor (Ldlr), Apolipoprotein E (ApoE) receptor 2 (Apoer2/Lrp8), very-LDL receptor (Vldlr), LDL receptor-related protein 1 (Lrp1), Lrp1b, Lrp2/Megalin and multiple epidermal growth factor (EGF) repeat containing protein 7 (Megf7/Lrp4; Dieckmann et al., 2010). Structurally, the extracellular domain (ECD) of each of the core LDL receptor family members is composed of a combination of two types of conserved domains: (1) ligand binding-type repeat domains (LBDs); and (2) EGF-precursor homology domains. The amino-terminal LBD domain confers ligand specificity, consisting of cysteine-rich complement-type ligand binding-type repeats (LBRs, sometimes called type A repeats). The EGF-precursor domains participate in the pH-dependent release of bound ligands after endocytosis and contain a mixture of EGF receptor-like repeats (EGF-repeats) and YWTD (Tyr-Trp-Thr-Asp) β-propeller repeats (Beglova and Blacklow, 2005; Andersen et al., 2013; reviewed in Li et al., 2001). The intracellular domain is less conserved between the family members, but each of the core members contain at least one NPxY (Asn-Pro-X-Tyr) motif that functions in protein interaction/signal transduction (Trommsdorff et al., 1998; Howell et al., 1999; Gotthardt et al., 2000) and endocytosis (Chen et al., 1990).

The smaller receptors within the LDL receptor family, Ldlr, Vldlr and Apoer2, contain only one EGF-precursor domain and have a juxtamembraneous domain rich in serine and threonine residues, which serve as sites for O-linked glycosylation (Kingsley et al., 1986; Sakai et al., 1994; Christie et al., 1996; Kim et al., 1996). This O-linked sugar (OLS) domain is alternatively spliced in both Apoer2 and Vldlr (Sakai et al., 1994; Kim et al., 1997; Clatworthy et al., 1999), and inclusion of the OLS-domain hinders the proteolytic processing of the receptors (Magrané et al., 1999; May et al., 2003; Wasser et al., 2014). However, for Apoer2 it was shown that exclusion of the OLS-domain produces “cleavage-resistant” Apoer2 splice variants, as the OLS-domain is likely the site of the initial extracellular cleavage that precedes further processing by γ-secretase (Wasser et al., 2014).

Additional somewhat distant members are Lrp5 and Lrp6 as well as the Sortilin-related receptor with LDLR class A repeats (SorLA; Figure 1). Lrp5 and Lrp6 (called arrow in *D. melanogaster*) encode four EGF-precursor domains but lack N-terminal LBDs and intracellular NPxY-motifs (Brown et al., 1998; Nakagawa et al., 1998; Wehrli et al., 2000). SorLA (SorL1/LR11/Lrp11), with multiple LBDs and one EGF-precursor domain, is a hybrid-LDL receptor family member in that it has an additional Vps10p-sorting domain and Fibronectin repeats (Jacobsen et al., 1996). In addition, SorLA has one NPxY-related retromer binding motif (FANSHY; Phe-Ala-Asn-Ser-His-Tyr; Fjorback et al., 2012). Containing three to five LBRs and no other typical LDL receptor domains, the most distant relatives are Lrad3 (Ranganathan et al., 2011) as well as Lrp3 (Ishii et al., 1998), Lrp10 (murine Lrp9; Sugiyama et al., 2000) and Lrp12 (ST7/Mg13; Battle et al., 2003), which have two additional CUB domains.

### Genetics

Despite the high degree of homology between the receptors and the overlapping expression pattern and function, the majority of these receptors are indispensable for survival or proper brain function. In fact, deletion of Lrp1 (Herz et al., 1992), Lrp1b (Dietrich et al., 2014), Lrp2 (Willnow et al., 1996), Lrp4 (Weatherbee et al., 2006) or Lrp6 (Pinson et al., 2000) in the mouse lead to embryonic or postnatal death with complete or high penetrance. While mice lacking Lrp5 (Fujino et al., 2003), Ldlr (Shimada et al., 1996), Apoer2 or Vldlr (Trommsdorff et al., 1999), or the distant member SorLA (Andersen et al., 2005) survive, they all have abnormalities in cholesterol homeostasis and/or brain development. Of the most distant relatives, gene silencing of Lrp12 leads to defects in brain lamination (Grote et al., 2016), yet to date *in vivo* knockouts or knockdowns of the more distant members Lrp3, Lrp10 and Lrad3 have not been reported.

## Lipoprotein Metabolism and Alzheimer’S Disease

One percent of all AD cases are early onset (EOAD) generally manifesting from mutations in APP or APP processing genes and leading to increased production of the toxic APP cleavage product, amyloid β (Aβ). The other 99% of cases are late-onset AD (LOAD) with increased Aβ-levels and deposition that are apparently independent from EOAD-like mutations in APP/APP processing genes. Instead, the leading cause in LOAD appears to be an imbalance between Aβ production and clearance from the brain (Weller et al., 2008; Mawuenyega et al., 2010). Thus, it is important to understand the various mechanisms by which LDL receptor family members and their ligands clear Aβ.

Aside from age, the most important risk modifier for developing LOAD is ApoE (Corder et al., 1993). ApoE is a major cholesterol transporter in the brain and in the circulation. In humans there are three ApoE alleles: ε2, ε3, and ε4 (ApoE2, 3 and 4, respectively). ApoE3 is the most abundant allele and understood as the neutral isoform with regards to AD-physiology, the least abundant isoform ApoE2 appears to be protective against AD (Corder et al., 1994; Conejero-Goldberg et al., 2014). Importantly, the ε4 allele of ApoE (ApoE4) dramatically reduces the age of AD onset and is carried by >50% of those afflicted with the disease (Corder et al., 1993), despite an allele frequency of only ~15% in the general population (Utermann et al., 1980). Therefore ApoE4 is the most prevalent, biomedically important risk allele for LOAD.

The brain is the most cholesterol-rich organ, containing approximately 25%–30% of the body’s total cholesterol (Dietschy and Turley, 2001), and high serum cholesterol levels correlate with cognitive impairment and AD (Zambón et al., 2010; Di Paolo and Kim, 2011). Interestingly, evidence from *in vivo* studies suggests that altered serum cholesterol levels affect the processing of APP as well as the neurotoxicity and clearance of Aβ (Reed et al., 2014). Despite this, the role of cholesterol metabolism in the pathogenesis of AD is not well understood.

The cholesterol metabolism link to AD pathogenesis is further supported by additional genome-wide association studies that implicate other apolipoproteins and their receptors as AD risk factors. In addition to ApoE, a variety of SNPs in ApoJ/Clusterin from several populations are associated with LOAD (Harold et al., 2009; Bagyinszky et al., 2014). Other apolipoprotein polymorphisms associated with AD have been reported in ApoA-I (Shibata et al., 2013), ApoA-IV (Császár et al., 1997), ApoC-I (Ki et al., 2002; Zhou et al., 2014; Shang et al., 2015), ApoC-II (Schellenberg et al., 1992), ApoC-III (Sun et al., 2005) and ApoD (Shibata et al., 2013). Among the LDL receptor family members, mutations in SorLA (Meng et al., 2007; Bagyinszky et al., 2014) appear to impart the most dramatic risk for developing AD. Aside from SorLA, Lrp1 (Kang et al., 1997), Lrp1b (Shang et al., 2015), Lrp2 (Wang et al., 2011), Lrp4 (Vargas et al., 2010), Lrp6 (De Ferrari et al., 2007) and Apoer2 (Ma et al., 2002) have been associated with AD risk. Furthermore, a non-LDL receptor family member, Trem2 (triggering receptor expressed on myeloid cells 2), is an alternative receptor for apolipoproteins, including ApoE and ApoJ/Clusterin, and has recently been identified as high risk factor for LOAD (Jin et al., 2015). In sum, cholesterol metabolism and the homeostasis/signaling of lipoprotein receptors and their ligands appear to be inextricably linked to the pathogenesis of LOAD.

With diverse functions including gathering nutrients and clearing toxic, useless debris from the extracellular space, as well as mediating intracellular trafficking/signaling and even transcription, the indispensable nature of many of the lipoprotein receptors is not surprising. Most of these receptors play some part in APP processing or clearance of Aβ, affecting the balance between Aβ-production and clearance. Understanding how these lipoprotein receptors and their ligands influence the homeostasis of Aβ production/clearance individually, as well as in unison, will prove crucial for not only elucidating mechanisms of AD pathogenesis, but also the design of potential therapeutic interventions to counteract the disease. In this chapter, we will focus on lipoprotein receptors and their role in AD pathogenesis through regulating APP processing and Aβ clearance.

## Ldlr

### Structure and General Physiological Properties

Ldlr, the founding member of the LDL receptor family, is ubiquitously expressed throughout the body, where it plays a key role in regulating cholesterol homeostasis (reviewed in Go and Mani, 2012). The receptor clusters after binding cholesterol-rich LDL particles and mediates cholesterol uptake through clathrin-mediated endocytosis of the lipoprotein-bound receptor (reviewed in Brown and Goldstein, 1979). Mutations in the *Ldlr* gene are responsible for familial hypercholesterolemia (FH), a disease in which Ldlr function is impaired, leading to increased plasma cholesterol concentrations and causing premature cardiovascular disease (Hobbs et al., 1990; Fass et al., 1997).

### Genetics

While impaired Ldlr function in humans leads to elevated plasma cholesterol and premature cardiovascular disease due to reduced uptake of cholesterol-rich LDLs (Hobbs et al., 1990; Fass et al., 1997), the effect in mice is similar yet less severe (Ishibashi et al., 1993; Osono et al., 1995). In the CNS, where Ldlr is expressed higher in astrocytes than in neurons, Ldlr also plays a role in cholesterol homeostasis in the brain. Ldlr knockout mice display some synaptic and learning deficiencies (Mulder et al., 2004, 2007; de Oliveira et al., 2011, 2013, 2014; Moreira et al., 2012). Interestingly, murine ApoE expression is elevated in the CSF of mice lacking Ldlr, and this phenotype is even more dramatic in mice carrying the human ApoE3 and ApoE4 isoforms of ApoE (Fryer et al., 2005). Ldlr deficiency also leads to elevated neuroinflammatory responses and oxidative stress (Thirumangalakudi et al., 2008; Katsouri and Georgopoulos, 2011), which might be further exacerbated by a high cholesterol diet (Ettcheto et al., 2015).

### Biochemistry and Cellular Function

As cholesterol metabolism is linked to AD and regulated by Ldlr, Ldlr knockout mice have been used as a model organism to study the interplay between cholesterol and Aβ-deposition in several studies. While Ldlr has no known direct or indirect interaction with APP or APP processing, Ldlr binds to Aβ and mediates its clearance by degradation in astrocytes, but does not alter APP processing (Kim et al., 2009). Ldlr knockout mice are more susceptible to Aβ-induced neurotoxicity, when Aβ is injected into the hippocampus (de Oliveira et al., 2014). Aβ-deposition is exacerbated with Ldlr-deficiency in AD mice (Tg2576 and APP/PS1; Cao et al., 2006; Katsouri and Georgopoulos, 2011) and is attenuated with Ldlr overexpression on an APP/PS1 background due to enhanced clearance (Kim et al., 2009). The additional knockout of ApoE does not affect the Aβ levels in Ldlr-deficient AD mice (APP/PS1; Katsouri and Georgopoulos, 2011), and this was confirmed by an *in vitro* study in astrocytes demonstrating that the clearance of Aβ is independent of ApoE (Basak et al., 2012). This suggests that the Ldlr-dependent glia response in Aβ-clearance is independent of ApoE despite Ldlr being a strong ApoE receptor (Katsouri and Georgopoulos, 2011; Basak et al., 2012). Nonetheless, Castellano et al. (2011) showed that Aβ turnover in the mouse brain *in vivo* is strongly dependent upon ApoE isoform, indicating that other mechanisms besides Ldlr-mediated Aβ removal are responsible for Aβ homeostasis in the intact brain.

## Lrp1

### Structure and General Physiological Properties

The second receptor identified in the LDL receptor family, Lrp1 (Herz et al., 1988) is one of the largest (~600 kDa) and most versatile members as it is known to bind over 100 different ligands (Herz and Strickland, 2001; Gonias and Campana, 2014). Lrp1 can be processed by the same enzymes as APP: ADAM10 (Nakajima et al., 2013), BACE1 (von Arnim et al., 2005) and γ-secretase (May et al., 2002; May and Herz, 2003; Zurhove et al., 2008). The sequential processing of Lrp1 first produces a soluble Lrp1-ECD, followed by a γ-secretase-mediated release of the Lrp1-ICD (May et al., 2002). The Lrp-ECD is capable of binding Lrp1 ligands (Quinn et al., 1997), and the Lrp1-ICD can translocate to the nucleus and regulate gene transcription (Zurhove et al., 2008). Of note, this Lrp1-ICD-mediated transcriptional regulation might be relevant to neuroinflammation (Zurhove et al., 2008), which is emerging as a common factor in many neuropathological conditions including AD (Heneka et al., 2015; Chen et al., 2016). Lrp1 also undergoes rapid, constitutive recycling; despite the two NPxY motifs in the Lrp1 cytoplasmic tail, a YxxL motif in the intracellular domain of Lrp1 is the dominant and main mediator of Lrp1 endocytosis—unlike other lipoprotein receptors, where the NPxY motifs mediate this process (Li et al., 2000). In addition to the liver and vasculature, Lrp1 is highly expressed in the brain (Rebeck et al., 1993) where it plays essential roles in signal transduction and endocytosis (Herz and Strickland, 2001; May et al., 2004). During brain development, it modulates radial glia stem cell proliferation, survival and differentiation (Safina et al., 2016). Importantly, Lrp1 can regulate the amyloidogenic processing of APP as well as the clearance of Aβ, which implicates Lrp1 as a key participant in the pathogenesis of AD (Kounnas et al., 1995; Ulery et al., 2000; Van Uden et al., 2000).

### Genetics

Global Lrp1 knockout mice are embryonically lethal (Herz et al., 1992, 1993). Lrp1 gene polymorphisms have been associated with a premature risk of cardiovascular disease in patients with familial hypercholesterolemia/FH (Aledo et al., 2012) and abnormal inflammatory responses in fibroblasts (Klar et al., 2015).

### Biochemistry and Cellular Function

Lrp1 directly interacts with APP extracellularly and regulates the localization and processing of APP (Kounnas et al., 1995). In several cell lines, depletion of the rapidly recycling Lrp1 reduced Aβ production (Ulery et al., 2000; Pietrzik et al., 2002). *In vivo*, overexpression of a minireceptor of Lrp1 (EGF-precursor domain-II, TM-domain, and ICD-domain) in an AD mouse model (PDAPP) increased soluble brain Aβ (Zerbinatti et al., 2004); however, reduced levels of Lrp1 in hippocampal neurons of another AD mouse model (APP/PS1) had no effect on Aβ production (Xu et al., 2012).

The extracellular interaction of Lrp1 and APP only occurs with APP isoforms containing the Kunitz protease inhibitor (KPI) domain and promotes the internalization of APP (Kounnas et al., 1995; Billnitzer et al., 2013). The KPI domain is present in the longer APP isoforms (APP_770_ and APP_751_) but not in the shortest, principally neuronal isoform (APP_695_), which is the dominant isoform in the brain (reviewed in Nalivaeva and Turner, 2013). This Lrp1-APP interaction can be blocked with the chaperone and Ldlr receptor family member antagonist, RAP (receptor-associated protein; Kounnas et al., 1995; Kinoshita et al., 2001). In hippocampal neurons, RAP treatment inhibited axonal branching due to increased APP on the cell surface that signals via complex formation with Fe65 and Mena (Ikin et al., 2007; Billnitzer et al., 2013). In APP knockout neurons, which have increased axonal branching compared to wildtype, RAP treatment had an additive Erk2-associated effect on branching (Billnitzer et al., 2013).

Intracellular interactions with APP and Lrp1 also appear important in modulating the amyloidogenic processing of APP. Both Fe65 and Dab1 interact with Lrp1 NPxY motifs and modify intracellular signal transduction (Trommsdorff et al., 1998; Gotthardt et al., 2000; Kinoshita et al., 2001; Pietrzik et al., 2004). These adaptors also bind APP (Fiore et al., 1995; Trommsdorff et al., 1998). The cytoplasmic adaptor protein, Fe65, links APP to Lrp1 and enhances amyloidogenic processing of APP (Pietrzik et al., 2002; Kinoshita et al., 2003; Yoon et al., 2005; Klug et al., 2011). Dab1 can interfere with this Lrp1/Fe65/APP complex by competing with Fe65 for Lrp1 binding, thereby reducing amyloidogenic APP processing (Kwon et al., 2010). Of note, the ICD of APP along with Fe65 translocates to the nucleus where it suppresses Lrp1 transcription (Liu et al., 2007). APP and Lrp1 also share other cytoplasmic interactions, one of which is with the endosomal sorting nexin 17 (Snx17). Snx17 interacts with the NPxY motifs in Lrp1 and APP to regulate their recycling from early endosomes back to the cell surface (Lee et al., 2008; Donoso et al., 2009; Farfán et al., 2013).

Despite promoting neuronal Aβ production, Lrp1 participates in Aβ clearance (reviewed in Kanekiyo and Bu, 2014). Lrp1 binds Aβ, with higher affinity for Aβ_40_ than Aβ_42_ (Shibata et al., 2000; Storck et al., 2016). Within the brain, Lrp1 endocytoses Aβ from the extracellular space and directs it to the lysosome for degradation (Kanekiyo et al., 2013). Lrp1 is also expressed in astrocytes and microglia where it is involved in Aβ-clearance (reviewed in Ries and Sastre, 2016). Another major Aβ clearance mechanism involves the transcytosis of Aβ from the brain to the circulation via the BBB (Marques et al., 2013). Lrp1 gene silencing reduced the clearance of intracerebroventricularly-injected Aβ across the BBB in wildtype mice (Jaeger et al., 2009). Furthermore, an endothelial (brain and choroid plexus)-specific Lrp1 knockout revealed that Lrp1 preferentially clears Aβ_40_, as these mice accumulated Aβ_40_ faster and demonstrated reduced spatial memory (Storck et al., 2016), which is a common phenotype observed with high levels of Aβ. Moreover, Lrp1 cleavage by ADAM10 has opposing effects as well; whereas soluble Lrp1 in the brain inhibits Aβ clearance, in the periphery it could provide a sink for Aβ monomers. Inhibition of ADAM10 reduces Lrp1 ectodomain shedding, thereby promoting Aβ-clearance across the BBB, especially Aβ_40_ (Shackleton et al., 2016); however, ADAM10 cleavage of Lrp1 also leads to the segregation of soluble Lrp1 into the periphery where it has been described to prevent the reentering of Aβ monomers into the brain (Sagare et al., 2007). Recently it was found that another AD risk gene, PICALM, plays a central role in BBB transcytosis of Aβ, and it has been reported that extracellular binding of Aβ to Lrp1 induces an intracellular conformational change allowing for PICALM binding and endocytosis of the entire complex (Zhao et al., 2015).

**Figure 2:**
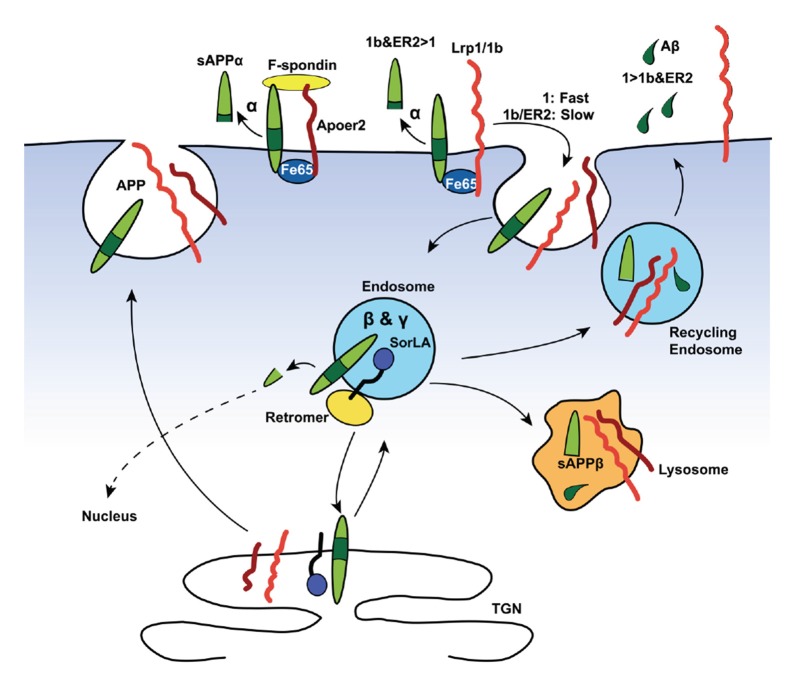
**Lipoprotein receptors modulate amyloid precursor protein (APP) trafficking and processing in neurons.** Neurons are the major source of Aβ (depicted as green droplets) in the brain. APP (green), all core LDL receptor family members as well as the more distant member SorLA contains at least one NPxY-motif, which acts as a docking site for PTB-domains of intracellular adaptor/scaffold proteins. Both Fe65 and Dab1 bind APP, as well as a number of LDL receptor family members (red and orange), via their PTB-domains. The simultaneous binding of these intracellular adaptor/scaffolding proteins to the NPxY motifs of APP and LDL receptors coordinate their intracellular trafficking, thus regulating APP localization and processing. The adapter/scaffold proteins control the speed of endocytosis of the receptors in that Fe65 and Dab1 binding to APP masks the endocytosis signal of APP, resulting in the surface retention of APP. This increases the exposure of APP to α-secretase (α), which cleaves APP inside the Aβ region (dark green) to release a soluble APPα (sAPPα) fragment and ultimately preventing the production of Aβ. Importantly, Lrp1 and Lrp1b (both orange in the diagram) have drastically different rates of endocytosis, with the internalization rate of Lrp1 exceeding that of Lrp1b by many-fold. Both bind Fe65, connecting them in a complex APP, and have opposite effects on APP processing. The fast endocytosis rate of Lrp1 increases the exposure of APP to the endosomal β- (BACE1, β) and γ-secretase (γ), producing Aβ (green tears) and soluble APPβ (sAPPβ) fragment. Another intraendosomal sorting receptor of the LDL receptor family, SorLA, can bind and reroute receptors from the endosome back to the trans-Golgi network (TGN), where it is either sequestered, sorted back to the cell surface, or sent to the lysosome for degradation. Apoer2, which also recycles slowly, binds Fe65 via its NPxY-motif, promoting APP surface stability and decrease amyloidogenic processing. Additionally, simultaneous binding of the secreted, extracellular ligand, F-spondin, to the ECDs of APP and Apoer2 also promotes APP stability at the surface.

**Figure 3:**
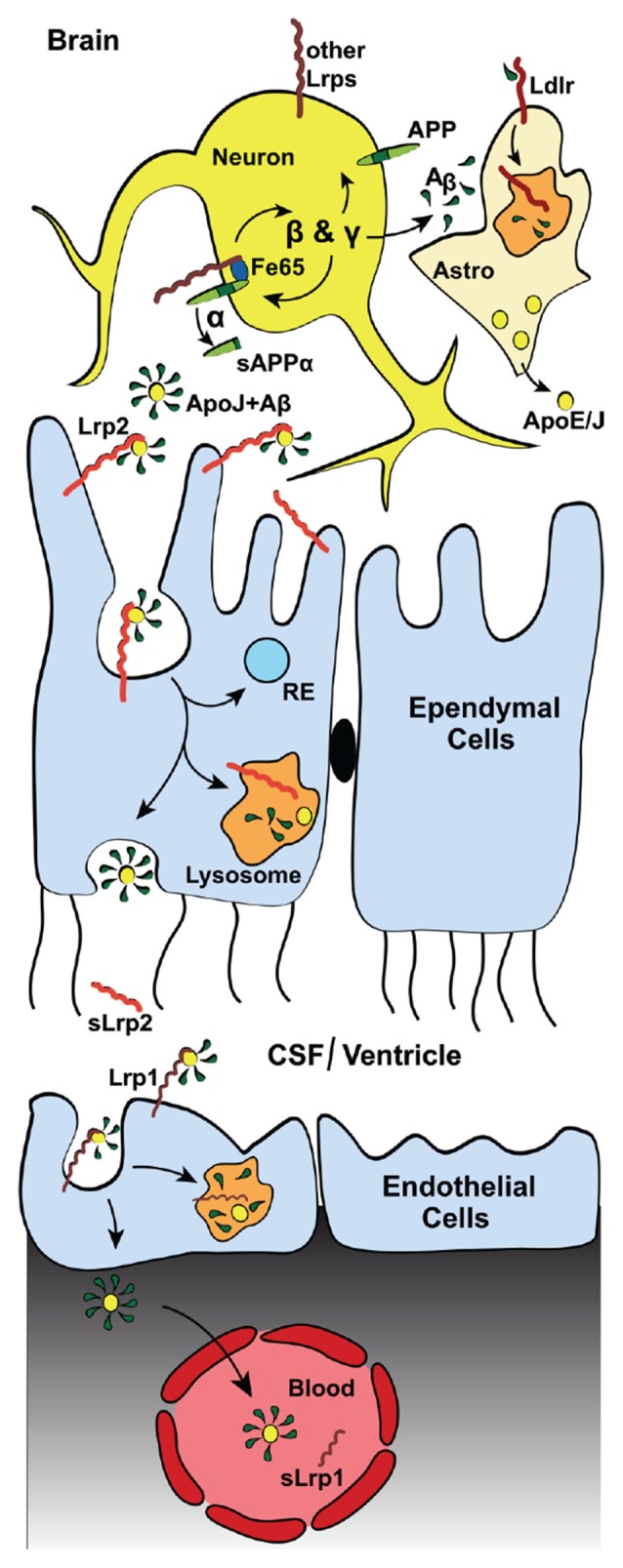
**Lrp2 mediates Aβ-clearance via the blood cerebrospinal fluid (CSF) barrier (BCSFB).** Diagram depicting the Lrp2-mediated clearance of interstitial Aβ through the cerebral spinal fluid (CSF) into the blood. In addition to direct astrocytic Lrp2 clearance of Aβ, Lrp2 expressed in the ependymal cells of the choroid plexus also facilitate Aβ removal. The choroid plexus functions to produce and filter CSF. This filtration removes metabolic waste, excess neurotransmitters and foreign/toxic particles, such as Aβ, which is mainly produced by neurons (see Figure 2). Apolipoproteins, such as ApoE and ApoJ/Clusterin (yellow dots), mainly secreted from astrocytes (“Astro”), bind circulating interstitial Aβ. These Aβ-laden apolipoproteins then bind lipoprotein receptors (red) and mediate their cellular uptake. ApoJ/Clusterin is eliminated rapidly across the BCSFB by ependymal Lrp2 (light red), facilitating the clearance of Aβ via lysosomal degradation in ependymal cells and subsequent exocytosis into the CSF, where soluble Lrp2 (sLrp2) has been detected (Spuch et al., 2015). BACE1 is the enzyme that processes Lrp2 and Lrp1 to release sLrp2 and sLrp1, respectively. BACE1 is also found in the choroid plexus (Crossgrove et al., 2007; Liu et al., 2013). Other lipoprotein receptors (dark red, most notably Lrp1) then transport Aβ and the apolipoproteins across the endothelial cells from the CSF to the blood vessels of the choroid plexus. sLrp1 can also be detected in plasma, albeit its origin there is mainly peripheral.

Importantly, both the Vldlr- and Lrp1-mediated Aβ clearance mechanisms via the BBB are differentially slowed down by ApoE-isoforms: ApoE4 > ApoE2 or ApoE3 (Deane et al., 2008). Besides clearance of Aβ, Lrp1 can compete with APP for BACE1 (von Einem et al., 2010) and γ-secretase (Lleó et al., 2005) cleavage. Taken together, it appears that Lrp1 contributes to the Aβ-homeostasis in two opposing ways: whereas Lrp1 promotes intraneuronal APP processing towards Aβ (Figure 2), Lrp1 also provides an important clearance mechanism of Aβ across the BBB and/or BCSFB (Figure 3).

## Lrp1b (LRP-DIT)

### Structure and General Physiological Properties

Lrp1b is very similar to Lrp1 in overall structure and sequence (~59% identical). Where Lrp1b differs most from Lrp1 is an extra LBR in the ECD and a 33 amino acid insert in the ICD (Liu et al., 2000). Lrp1b was first associated with tumorigenesis, but is also highly expressed in the adult brain (Liu et al., 2000; Haas et al., 2011) and retains APP at the cell surface reducing Aβ production (Cam et al., 2004).

### Genetics

Mutations in Lrp1b are associated with multiple different types of cancer (Liu et al., 2000; Langbein et al., 2002; Sonoda et al., 2004), including gliomas (Roversi et al., 2006). Lrp1b-deficiency leads to embryonal lethality (Dietrich et al., 2010). Like Lrp4 knockins expressing a truncated ECD (see “Lrp4” Section for details), a similar truncation of Lrp1b allows animals to survive, be fertile and develop mostly normal. However, in contrast to Lrp4-ECD (Pohlkamp et al., 2015) mice, synaptic plasticity in hippocampal field recording is not affected in Lrp1b-ECD mice (Marschang et al., 2004).

### Biochemistry and Cellular Function

Lrp1b binds to fibrinogen and ApoE carrying proteins (Haas et al., 2011). In total, Lrp1 and Lrp1b share numerous ligands. Lrp1b also binds APP at the extracellular KPI-containing domain (Cam et al., 2004). With an internalization rate of more than 10 min for Lrp1b, the rate of endocytosis is much slower than Lrp1, which has a rate of less than 30 s (Liu et al., 2001). In contrast to overexpression of Lrp1 in a cell culture system, overexpression of Lrp1b increased APP surface expression, resulting in enhanced non-amyloidogenic α-secretase cleavage and reduced Aβ production (Cam et al., 2004). Based on these *in vitro* findings, a model for the Lrp1- vs. Lrp1b-effect on APP processing was proposed by Wagner and Pietrzik (2012), where fast Lrp1 uptake shifts APP processing from α-cleavage towards the endosomal toxic β- and γ-cleavage-pathway, whereas Lrp1b-APP interaction results in prolonged surface time and increased α-cleavage of APP (Figure 2). However, it is important to note that while Lrp1, not Lrp1b, is likely to promote intracellular Aβ-production, it is conversely important for Aβ-clearance across the BBB.

## Apoer2 (Lrp8) and Vldlr

### Structure, General Physiological Properties and Genetics

Both Apoer2 and Vldlr are quite similar in size and domain composition to Ldlr (Figure 1; Kim et al., 1996). The sequence identity between Vldlr and Apoer2 is approximately 50% (Kim et al., 1996 and reviewed in Reddy et al., 2011). Apoer2 has seven ligand-binding repeats, one less than Vldr, and contains a unique alternatively-spliced proline-rich domain not found in Vldlr (Kim et al., 1997; Clatworthy et al., 1999; Sun and Soutar, 1999). In the brain, Apoer2 only contains five ligand-binding domains due to alternative-splicing of exon 5 (Kim et al., 1997; Clatworthy et al., 1999; Sun and Soutar, 1999). The site of least homology between the Apoer2 and Vldlr is the OLS domain (Kim et al., 1996). As mentioned above, the OLS domain is alternatively-spliced in both receptors. For both receptors, splice variants containing the OLS domain are highly glycosylated, and this glycosylation inhibits proteolytic processing (Magrané et al., 1999; May et al., 2003; Wasser et al., 2014). For Vldlr, splice variants lacking this glycosylated domain undergo rapid proteolytic cleavage (Magrané et al., 1999). Unlike Vldlr, the OLS domain is required for the initial extracellular cleavage of Apoer2 (presumably due to loss of the extracellular cleavage site), so Apoer2 variants lacking the OLS domain are actually resistant to proteolysis (Wasser et al., 2014).

Apoer2 and Vldlr are almost exclusively expressed in the brain where they act as receptors not only for ApoE but also for the neuromodulator Reelin (D’Arcangelo et al., 1999; Trommsdorff et al., 1999). Ligand binding increases the proteolytic processing of both receptors (Hoe and Rebeck, 2005). The proteolytic fragments of Apoer2 can inhibit further signaling, whereby the soluble ECD fragment acts as a dominant negative receptor (Koch et al., 2002) and the released ICD translocates to the nucleus and represses Reelin transcription (Balmaceda et al., 2014; Telese et al., 2015).

The signaling initiated by Reelin binding to Apoer2 and Vldlr plays essential roles during the development of the CNS and neuronal function through adulthood (Förster et al., 2010). During development, Reelin expressed and secreted from Cajal-Retzius cells modulates the cytoskeleton and mobility of migrating neurons (Frotscher et al., 2009) and ensures proper cortical, hippocampal and cerebellar lamination (Trommsdorff et al., 1999).

Apoer2 and Vldlr double knockout leads to a phenotype comparable to Reelin or Dab1 deficiency: mice develop strong ataxia, a smaller cerebellum, and defective lamination of cerebellum, cortex and hippocampus (Trommsdorff et al., 1999).

Cortical Cajal-Retzius cells die out after birth and the amount of hippocampal Cajal-Retzius cells dramatically thins out later during postnatal hippocampal maturation (Chowdhury et al., 2010). In total, the expression pattern changes so that in the cortex and hippocampus Reelin is now expressed in a more distributed fashion, mainly by subtypes of GABAergic interneurons (Drakew et al., 1998; Pesold et al., 1998; Pohlkamp et al., 2014). Besides neuronal migration, Reelin-signaling plays parts in both axo- (Leemhuis et al., 2010) and dendritogenesis (Assadi et al., 2003; Niu et al., 2004; Jossin and Goffinet, 2007; Zhang et al., 2007; Kawauchi and Hoshino, 2008; Matsuki et al., 2008; Chai et al., 2009; Ventruti et al., 2011) as well as synapse formation and function (Glantz and Lewis, 2000; Sinagra et al., 2005; Groc et al., 2007; Qiu and Weeber, 2007; Niu et al., 2008; Campo et al., 2009; Dumanis et al., 2011; Hellwig et al., 2011; Bal et al., 2013). In the adult brain, Reelin regulates synaptic function, plasticity and spatial learning and fear memory (Weeber et al., 2002; Beffert et al., 2005; Herz and Chen, 2006; Wasser et al., 2014).

Apoer2 and Vldlr bind Reelin and cluster together resulting in the phosphorylation of Dab1 and Src-kinase-mediated phosphorylation of NR2 subunits of the NMDA receptor (Hiesberger et al., 1999; Arnaud et al., 2003; Bock and Herz, 2003; Strasser et al., 2004), which requires a unique 59-amino acid insert in the Apoer2 cytoplasmic tail through direct interaction with PSD-95 (Beffert et al., 2005). Reelin-mediated NMDAR phosphorylation increases Ca^2+^-influx through NMDAR, resulting in increased activation of cAMP-response element binding protein (CREB; Chen et al., 2005) and the potent enhancement of long-term potentiation (LTP; Weeber et al., 2002). Hippocampal LTP is modestly reduced or severely perturbed in mice lacking Vldlr or Apoer2, respectively, and LTP is not enhanced by acute Reelin treatment in either mutant (Weeber et al., 2002).

There are several lines of evidence that implicate Reelin signaling as protective against AD pathogenesis. First, Reelin-signaling can counteract Aβ-induced synaptic suppression (Durakoglugil et al., 2009) by enhancing synaptic LTP, an effect that requires a unique alternatively spliced exon in the ICD of Apoer2 (Beffert et al., 2005). Interestingly, the AD-risk factor ApoE4 actually prevents this protective effect by sequestering the ApoE receptors along with other synaptic receptors in the endosome (Chen et al., 2010), and postnatal loss of Reelin exacerbates the cognitive deficits in AD mouse model (Lane-Donovan et al., 2015). In AD mice, Apoer2 and its ligand Reelin are localized in fine granular structures and reactive astrocytes surrounding Aβ plaques (Wirths et al., 2001; Motoi et al., 2004). Furthermore, both humans with AD and a transgenic AD mouse model have higher expression of the Apoer2 splice variant that lacks the alternatively spliced CTD, which would be predicted to impair the Reelin-mediated suppression of Aβ-toxicity (Hinrich et al., 2016). Treating these AD mice with antisense oligonucleotides designed to increase the inclusion of the alternatively spliced proline-rich domain in Apoer2 restored the expression of the functional Apoer2 variant and rescued their AD-related memory deficits (Hinrich et al., 2016).

### Biochemistry and Cellular Function

Both Apoer2 and Vldlr interact with APP-binding proteins and influence the amyloidogenic processing of APP (reviewed Hoe and Rebeck, 2008; Marzolo and Bu, 2009; Wagner and Pietrzik, 2012; Lane-Donovan et al., 2014). Of the two receptors, Apoer2 interacts with a larger number of APP-binding proteins. Both APP and Apoer2 bind F-spondin (Ho and Südhof, 2004; Hoe et al., 2005) and Reelin (Hoe et al., 2009) extracellularly, as well as the intracellular adaptor proteins X11α/β (Borg et al., 1996; He et al., 2007), Fe65 (Fiore et al., 1995; Borg et al., 1996; Hoe et al., 2006a), Snx17 (Lee et al., 2008; Sotelo et al., 2014), Dab1 (Homayouni et al., 1999; Howell et al., 1999), and Dab2 (Cuitino et al., 2005; Lee et al., 2008). To date, Vldlr is known to directly interact with both Reelin and Fe65 (Dumanis et al., 2012) and immunoprecipitation results supported that Fe65 increases the interaction between APP and Vldlr *in vivo*, suggesting that Vldlr is involved in APP trafficking (Dumanis et al., 2012).

Ligand binding to Apoer2 induces homotypic clustering as well as clustering with other receptors, including APP (Divekar et al., 2014). The clustering of Apoer2 is weaker with ApoE binding compared to the clustering upon binding either Reelin or F-spondin (Divekar et al., 2014). ApoE inhibits γ-secretase cleavage of Apoer2 and APP (Irizarry et al., 2004; Hoe et al., 2006b), and ApoE3 imparted a greater inhibition than ApoE4 preventing the release of the Apoer2-ICD and APP intracellular domain (Hoe et al., 2006b). Interestingly, Apoer2-deficient mice express more ApoE and have elevated levels of the aggregation prone form of Aβ (Aβ_42_; Petit-Turcotte et al., 2005).

F-spondin is an extracellular ligand for both Apoer2 (Hoe et al., 2005) and APP (Ho and Südhof, 2004). This secreted extracellular protein, F-spondin, is composed of an amino-terminal Reelin and F-spondin domains followed by a thrombospondin domain, which contains six thrombospondin repeats (TSRs; reviewed in Feinstein and Klar, 2004). The central portion of the APP-ECD binds within the amino-terminal Reelin and F-spondin domains, while the LBD of Apoer2 binds the first four TSRs of F-spondin (Hoe et al., 2005). F-spondin stabilizes Apoer2 and APP at the cell surface, promoting α-cleavage of both proteins and reducing Aβ formation (Hoe et al., 2005). Of note, other LDL receptor family members-Vldlr, Lrp4 and Lrp2—also bind the first four TSRs of F-spondin (Zisman et al., 2007).

Like Lrp1, the NPxY domain of Apoer2 binds the cytosolic adaptor protein Fe65. While Lrp1 and Fe65 enhance Aβ production, Fe65 increases the interaction of APP and Apoer2 and decreases APP processing by stabilizing them at the cell surface (Hoe et al., 2006a). As Apoer2 and Lrp1 interact within the same region of Fe65, these two receptors may compete with each other for Fe65 binding and differentially influence APP processing (Hoe et al., 2006a). Dab1 also binds the NPxY motifs of Apoer2 and APP, and Aβ is decreased with Dab1 overexpression and increased in Dab1-deficient primary neurons (Hoe et al., 2006c).

Apoer2 directly interacts with APP extracellularly (Fuentealba et al., 2007). In Lrp1-deficient cells, Apoer2 promotes the cell surface retention of APP. This stabilization of APP requires cytoplasmic domain of Apoer2 (Fuentealba et al., 2007). Co-expression of Apoer2 with APP promotes APP surface expression and the lipid raft association of APP dependent on the Apoer2 CTD, but unexpectedly increased Aβ formation (Fuentealba et al., 2007). In contrast, X11α/β-binding to Apoer2 mediates ApoE induced endocytosis of APP and β-secretase resulting in APP processing and Aβ production (He et al., 2007), and Reelin can interrupt this interaction between X11α/β and Apoer2 (Minami et al., 2010), indicating another protective role of Reelin against Aβ toxicity.

## Lrp2 (Megalin/gp330)

### Structure and General Physiological Properties

Lrp2 is structurally very similar to Lrp1b and one of the most studied lipoprotein receptors in conjunction with AD. Similar to Lrp1, Lrp2 undergoes proteolytic processing to release the ECD followed by γ-secretase cleavage to release the ICD (Zou et al., 2004; Biemesderfer, 2006). The Lrp2-ICD contains sorting signals including three NPxY and a PPPSP motif that control Lrp2 surface expression specifically at cholesterol- and glycosphingolipid-rich regions (Marzolo et al., 2003). Besides binding to APP and ApoE, Lrp2 is also an important receptor for ApoJ/Clusterin, which is another genetic risk factor for AD. Lrp2 is expressed on endothelial cells of different organs, including capillaries in the brain and the ependymal cells of the choroid plexus, where it controls cholesterol homeostasis and Aβ-clearance (Willnow et al., 1996; Hammad et al., 1997; Chun et al., 1999; Bell et al., 2007). Besides its expression in endothelial and ependymal cells, Lrp2-expression has also been reported in dying neurons of postmortem brains of AD patients and cultured astrocytes (LaFerla et al., 1997; Bento-Abreu et al., 2008).

During neural tube formation and forebrain development Lrp2 is required for the dorsal to ventral gradient of the bone morphogenic protein 4 (BMP4) and sonic hedgehog (Shh). Lrp2 mediates endocytosis of Bmp4 for degradation and Bmp4 levels are increased in Lrp2-deficient mice (Spoelgen et al., 2005). Lrp2 is also a required co-receptor for Shh, ligand-binding induces a positive feedback loop and increased Shh-expression, thus Lrp2-deficiency leads to the loss of Shh expression in the ventral neuroepithelium (Christ et al., 2012). Finally, the loss of the Bmp4-Shh gradient in the neural tube causes holoprosencephaly, the failure of the brain to develop into two hemispheres (Spoelgen et al., 2005; Christ et al., 2012). Moreover, Shh and Lrp2 signaling regulates oligodendrocyte progenitor migration and proliferation in the optic nerve (Ortega et al., 2012) and glial cell specification during neural development (Wicher et al., 2005). The role of Lrp1 and Lrp2 in regulating neural stem cell and progenitor cell function has been reviewed in detail elsewhere (Auderset et al., 2016). However an implication of APP for these mechanisms has not been described.

### Genetics

Lrp2-deficient mice die shortly after birth due to respiratory insufficiency. Lrp2 function is critical during neural tube formation, as it acts to organize Shh-mediated forebrain development during neurulation (Christ et al., 2012). Besides malfunctioning of endothelial tissues including lung and kidney, Lrp2-deficiency in neuroepithelium leads to impaired proliferation and forebrain fusion (Willnow et al., 1996). Endothelial cell specific Lrp2 deletion leads to impaired Aβ-clearance, which is described in more detail in the next section.

### Biochemistry and Cellular Function

In the adult brain, Lrp2, facilitated by its ligand ApoJ/Clusterin, mediates Aβ clearance from the CSF (Hammad et al., 1997; Bell et al., 2007; Figure 3). As a part of the blood-CSF barrier (BCSFB), the choroid plexus takes part in the production and filtration of the CSF, including clearance of Aβ (Figure 2). Lrp2 is expressed within the choroid plexus, where it is sorted to the apical surface of ependymal cells within the lateral ventricles (Zheng et al., 1994; Chun et al., 1999; Willnow et al., 1999; Carro et al., 2005; Alvira-Botero and Carro, 2010). Despite a lack of AD pathology, mice lacking Lrp2 within these ependymal and endothelial cells display cognition deficits that mimic those in AD mice with elevated Aβ production (Dietrich et al., 2014). Of note, ApoJ/Clusterin also binds to Lrp1 (Gil et al., 2013), Vldlr, and Apoer2 (Andersen et al., 2003; Leeb et al., 2014) and alternative receptors Trem2 (Yeh et al., 2016) and Plexin A4 (Kang et al., 2016), yet it is not known how ApoJ/Clusterin interactions with the other LDL receptor family members affects AD pathology.

Lrp2 expression decreases with age, which goes along with a reduced clearance rate of Aβ (Carro et al., 2005). In brains of AD-patients, damaged neurons express more Lrp2 (LaFerla et al., 1997), and the transcription of Lrp2 mRNA is repressed by microRNA-146a (Zhang et al., 2016). Genetically, a single nucleotide polymorphism (SNP) in the Lrp2 promoter that reduces Lrp2 expression by 20% is considered a risk factor for AD (Vargas et al., 2010; Wang et al., 2011). Additionally, much like Lrp1, Lrp2 forms a complex with APP and Fe65 to control neurite branching and APP processing (Alvira-Botero et al., 2010).

## Lrp4 (Megf7)

### Structure and General Physiological Properties

One of the shorter members of the LDL receptor family, Lrp4, is critical for survival in that LRP4 knockout mice die after birth due to defects in the neuromuscular junction (NMJ; Weatherbee et al., 2006). Lrp4 is also involved in the development of both the kidneys and limbs as Lrp4 knockout mice display abnormal limb morphology and renal agenesis (Johnson et al., 2005; Simon-Chazottes et al., 2006; Karner et al., 2010; Tanahashi et al., 2016). Additionally, Lrp4 regulates chondrocyte and osteoblast homeostasis during cartilage and bone growth (respectively) through binding the ligands Wise/Sostdc1, Dickkopf and Sclerostin (Choi et al., 2009; Asai et al., 2014). As Lrp4-deficient mice die due to abnormal NMJ formation, Lrp4 plays a pivotal role during development at the NMJ where Lrp4 along with its ligand, the heparan-sulfate proteoglycan (HSPG) Agrin, and co-receptors muscle-specific tyrosine receptor kinase (MuSK) and APP act together to orchestrate NMJ formation (Kim et al., 2008; Zhang et al., 2008; Choi et al., 2013). The Lrp4 ligand, Agrin, similar to the Apoer2 and Vldlr ligand Reelin, which also interacts with APP, is a large extracellular matrix protein with multiple binding domains. On the muscle fiber membrane, MuSK and Lrp4 form a functional receptor complex for Agrin. Upon Agrin binding to Lrp4, MuSK is phosphorylated resulting in Rapsyn-dependent focal clustering of nicotinic Acetylcholine receptors (nAChR; Shen et al., 2014). Recent evidence suggests that these components, which are also expressed in the adult brain, also play a role in synaptic plasticity and/or AD pathogenesis (Glenner and Wong, 1984; Berzin et al., 2000; Gomez et al., 2014; Pohlkamp et al., 2015; Sun et al., 2016).

### Genetics

Deficiency in Lrp4, MuSK, Agrin, APP and APLP2, or the intracellular scaffold Rapsyn lead to neonatal lethality, due to failure to form NMJs (Gautam et al., 1999; Wang et al., 2005; Weatherbee et al., 2006). At central synapses, these components do not appear critical for synapse formation; however, a recent report demonstrated that Agrin, Lrp4 and MuSK act together on the astrocyte to control synaptic plasticity (Sun et al., 2016). Lrp4, like APP, is a substrate for ADAM10 secretase and γ-secretase and undergoes proteolytic processing by these enzymes to release soluble ECD and ICD fragments of Lrp4, respectively (Dietrich et al., 2014). Targeted expression of various Lrp4 truncations in mice revealed a differential dependence of membrane anchoring and the presence of the ICD for Lrp4-mediated mechanisms. Knockins expressing secreted Lrp4-ECD survive, but display impaired LTP and develop only partially functional NMJs with abnormal limb development. Alternatively, in mice expressing a membrane-anchored Lrp4 with deleted ICD limb development is only mildly affected and LTP is normal (Johnson et al., 2005; Choi et al., 2013; Pohlkamp et al., 2015).

Studies at the NMJ also revealed important insights how different members of the APP-family interact (Choi et al., 2013). In APP/APLP2 mutants, NMJ endplate patterning is severely impaired, whereas APLP1/APLP2 mutants develop normal endplate patterning with reduced size and apposition of pre- and postsynaptic specializations. APLP1 seems to be exclusively expressed in the neuronal ending of the NMJ whereas APP and APLP2 are present on both, the muscle and the neuronal sides (Klevanski et al., 2014). In addition, Fe65/Fe65L1 double knockout mice show severe motor impairments, NMJ pre- and postsynaptic appositions, and impaired hippocampal LTP (Strecker et al., 2016). Fe65 interacts with Apoer2, Vldlr, Lrp1, Lrp1b, Lrp2, but binding to Lrp4 has so far not been examined.

### Biochemistry and Cell Biology

**Figure 4:**
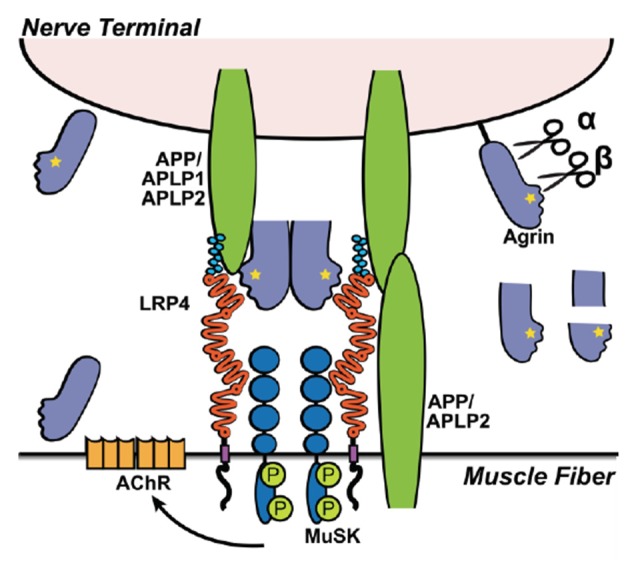
**Lrp4 and APP interaction during neuromuscular junction (NMJ) formation.** Illustration depicting the interaction of Lrp4, MuSK, Agrin and APP/APLP1/APLP2 in the formation of the NMJ. Agrin binds Lrp4 resulting in phosphorylation (P) of MuSK, which leads to the recruitment and clustering of acetylcholine receptors (AchRs). The recruitment of AChR to the NMJ depends on all components of the complex. Knockouts of Lrp4, MuSK, Agrin, or APP/APLP1/APLP2 result in defective NMJ formation and perinatal lethality. APP and its family members (APLP1 and APLP2) have redundant functions, allowing them to compensate if one is knocked out. APLP1 is expressed on the presynaptic motor neuron, whereas APLP2 and APP are expressed by both nerve cells and muscle cells. Double knockouts lacking both APP and APLP1 form functional NMJs and are viable, whereas APP^−/−^/APLP2^−/−^ and APLP1^−/−^/APLP2^−/−^ mice have severely defective neuromuscular synapses and high postnatal lethality, indicating that APLP2 is an essential component in NMJ formation, but APP and APLP1 together can partially compensate in the absence of APLP2. Agrin is expressed in both neurons and muscle cells, but each express different isoforms. Isoforms expressed by neurons differ from muscular Agrin by the Z+ splice insert (yellow star), required for Lrp4 binding (Zong et al., 2012) and NMJ-formation (Burgess et al., 1999). In addition, besides secreted Agrin, motorneurons express a TM Agrin, which is not required for NMJ-formation. Extracellular cleavage of Agrin (α- and β-sites) can be mediated by Neurotrypsin and other as-yet unidentified proteases (black) expressed at the muscle. While Agrin cleavage is required for proper NMJ maturation, Neurotrypsin-mediated cleavage of Agrin is not—despite the fact that Neurotrypsin overexpression leads to NMJ-failures (Bolliger et al., 2010). The small soluble Z+ containing C-terminal fragment (after β-cleavage) is sufficient to bind Lrp4 and induce AChR-clustering, but it is less efficient compared to full length Agrin or Agrin cleaved at the α-site, only (Zong et al., 2012).

On the muscle fiber membrane, MuSK and Lrp4 form a functional receptor complex for Agrin. Upon Agrin binding to Lrp4, MuSK is phosphorylated resulting in Rapsyn-dependent focal clustering of nAChR (Shen et al., 2014). APP, and presumably APLP2, present on the muscle fiber surface and along with APLP1 on the neuron, also binds to Lrp4 and Agrin, which is required for the localized clustering of AChR on the muscle fiber where nerves terminate to allow a functional NMJ to form (Kim et al., 2008; Choi et al., 2013; Figure 4). Interestingly, unlike Lrp1 and Lrp1b, *in vitro* experiments show that Lrp4 binding to APP does not require the KPI domain in APP (Choi et al., 2013).

Similar to the lipoprotein receptor ligand Reelin, multiple functions have been described for Agrin in shaping and maintaining neuronal activity in the brain. Agrin stimulates filopodia formation to allow structural plasticity (McCroskery et al., 2009) and inhibits astrocytic ATP release resulting in enhanced synaptic glutamate release (Sun et al., 2016). Agrin also regulates the strength of GABAergic synapses during network inactivation (Pribiag et al., 2014), reduces Aβ-levels (Rauch et al., 2011), and contributes to acetylcholine receptor clustering (Rauch et al., 2011). However, as of now, it is unknown if these functions require Lrp4-mediated endocytosis and trafficking. For example, Lrp4 does not require endocytic activity to promote NMJ formation (Willnow et al., 2012). Agrin binds not only Lrp4 but also to multiple other receptors and ligands such as heparin (Wallace, 1990), NCAM (Storms et al., 1996), Integrins (Martin and Sanes, 1997), α-dystroglycan (Bowe et al., 1994), Na^+^/K^+^ATPase (Hilgenberg et al., 2006) and notably APP (Choi et al., 2013). Moreover, presynaptic activity dependent release and postsynaptic activity- dependent activation of the protease Neurotrypsin regulates Agrin cleavage at α- and β-sites (Reif et al., 2007; Stephan et al., 2008; Gisler et al., 2013). Specifically the short C-terminal fragment of Agrin potentially promotes filopodia outgrowth via α-dystroglycan (Gisler et al., 2013).

Lrp4 also contributes to synaptic plasticity. Mice lacking Lrp4 or expressing a truncated Lrp4 retaining the ECD (Lrp4-ECD) in the brain have impaired hippocampal LTP and impaired memory (Gomez et al., 2014; Pohlkamp et al., 2015). Importantly, Sun et al. (2016) showed that the astrocyte-specific knockout of Lrp4 (using GFAP-Cre) extinguishes all brain Lrp4 expression and enhances the release of ATP from astrocytes, which may be causative for the described impairment in LTP. Of note, GFAP-Cre expression is not restricted to astrocytes and found in some neuronal populations as well. However, the authors also demonstrated that Agrin, by binding to Lrp4 and activating MuSK, controls the ATP release from astrocytes (Sun et al., 2016). The impaired LTP in Lrp4-ECD mice (Pohlkamp et al., 2015) suggests that anchoring of Lrp4 to the astrocytic membrane is required for normal synaptic potentiation. Neurons exclusively express the TM-Agrin (Neumann et al., 2001) that contains the alternatively spliced Z+ insert required for Lrp4 binding. TM-Agrin, by binding to Lrp4 could mediate a direct interaction of astrocytes and neurons. Furthermore, activity-driven neurotrypsin cleavage would allow the release of the Agrin C-terminal Lrp4-binding domain, which then can diffuse and bind to Lrp4/MuSK complexes on the astrocytic surface to control ATP release. It needs to be determined if this pathway requires APP or APLP1/2 in the complex, which are mainly/exclusively expressed by neurons. The astrocytic Agrin/Lrp4/MuSK complex together with APP or APLP2 on the neuronal surface might also be relevant for astrocyte-neuron interactions.

In the hippocampus, besides neurons, astrocytes express functional α7-type AchRs (Shen and Yakel, 2012), which is increased in the brain of AD-patients (Yu et al., 2005). Importantly Aβ binds to hippocampal α7AchR expressed on astrocytes, resulting in increased Ca^2+^ permeability (Pirttimaki et al., 2013). Activation of α7AchR on astrocytes triggers AMPA receptor recruitment to glutamatergic synapses, a mechanism also involved in converting silent synapses to functional ones (Wang et al., 2013a). At the NMJ Agrin/Lrp4/MuSK/APP complex formation appears to be required to effectively cluster AchRs. So far, however, astrocytic α7AchR function has not been shown to require the formation of an Agrin/Lrp4/MuSK/APP-complex. However, total AChR clustering in TM-Agrin knockout mouse brains, expressing only 20% of the Lrp4-binding Z+ Agrin form, is 4- to 5-fold reduced (Rauch et al., 2011).

Heparan sulfate proteoglycans (HSPG) inhibit BACE1 mediated APP cleavage (Scholefield et al., 2003). Thus, Agrin, as the major HSPG accumulating in plaques of AD-brains (Verbeek et al., 1999) might be a relevant inhibitor of BACE1. Agrin has also been described to be relevant for the function of the BBB (Rauch et al., 2011; Steiner et al., 2014). However, Aβ-clearance via Agrin and Lrp4 in astrocytes is unlikely, since in the neuron-specific TM-Agrin knockout, which expresses only 20% of Z+ Lrp4-interacting Agrin, Aβ clearance is not affected. By contrast, endothelial-specific knockout of Agrin does reduce Aβ-clearance (Rauch et al., 2011).

## Lrp5/6

### Structure and General Physiological Properties

Lrp5 and Lrp6 share 71% homology and are more distantly related members of the family. Despite encoding three LBRs and four EGF-precursor homology domains, compared to the core members, the domains appear in an inverse order with the ligand-binding domains adjacent to the TM segment rather than at the N-terminus. Additionally, their ICDs lack NPxY motifs. Both receptors have important functions in Wnt/β-catenin signaling, whereby Wnt and the Frizzled-receptors, mediate intracellular β-catenin translocation to the nucleus for transcriptional control of target gene expression (reviewed by Joiner et al., 2013). Similar to Lrp4, Lrp5 and Lrp6 are involved in bone growth (Lara-Castillo and Johnson, 2015), recently Lrp6 has also been suggested to have a role in AD and APP processing (De Ferrari et al., 2007).

### Genetics

Lrp5 deficiency causes osteoporosis and bone fracture in mice due to reduced osteoblast proliferation and low bone mass (Kato et al., 2002), and point mutations have been found in human patients with altered bone mass. Lrp5 knockout also leads to defects in cholesterol and glucose metabolism. Lrp5 and ApoE double knockout mice suffer from hypercholesterolemia, fat intolerance, and atherosclerosis (Fujino et al., 2003; Magoori et al., 2003). Mesenchymal specific Lrp5 and Lrp6 double mutants resembled β-catenin knockouts, with severe skeletal development defects (Joeng et al., 2011). Whereas Lrp5 deficiency primarily affects bone density, Lrp6 deficiency severely affects brain development. Lrp6 deletion leads to death after birth, similar to Wnt mutants they have a caudal truncation of the body axis, excess neural tissue, defects in neural tube closure, loss of paraxial mesoderm, and mid- and hindbrain defects (Pinson et al., 2000). A point mutation in an EGF repeat of Lrp6 causes coronary artery disease with high LDL-levels by affecting Wnt signaling (Mani et al., 2007). A SNP in a highly conserved region of LRP6, initially genetically associated with low bone mass, has now been associated with AD (De Ferrari et al., 2007).

### Biochemistry and Cellular Function

Wnt signaling via Lrp6 has been implicated in neuronal differentiation (Jeong et al., 2014), commissural axon guidance (Avilés and Stoeckli, 2016), and adult neurogenesis in the hippocampal niche (Schafer et al., 2015). Neuronal deletion of Lrp6 in the forebrain of the mouse leads to defects in synaptic integrity and memory formation. Furthermore crossing these mice with APP/PS1 mice led to increased APP processing to Aβ that in turn inhibited Wnt signaling, resulting in a synergistic effect on synaptic dysfunction (Liu et al., 2014). Wnt signaling is also compromised in brains of patients with AD (Liu et al., 2014).

## SorLA (Sorl1/LR11/LRP11)

### Structure and General Physiological Properties

SorLA is a hybrid-type receptor, as the only member of the LDL receptor family with a Vps10p (yeast vacuolar protein sorting 10 protein) domain and six Fibronectin repeats (Figure 1). SorLA is predominantly expressed in the brain, especially in neurons (Jacobsen et al., 1996; Yamazaki et al., 1996), where it acts as an intracellular sorting receptor transporting cargo, including APP, between different intracellular compartments in the cell (Andersen et al., 2005). In addition to familial mutations linked to AD (Meng et al., 2007), SorLA is reduced in postmortem AD brains (Scherzer et al., 2004) and in the CSF of AD patients (Ma et al., 2009).

### Genetics

Defective homeostasis of SorLA and its cargo disrupts cellular function and causes AD, atherosclerosis and obesity (Caglayan et al., 2014). In mice, SorLA knockout leads to increased Aβ-levels in the brain, whereas neuronal SorLA overexpression causes a redistribution of APP to the Golgi, which results in decreased Aβ production (Andersen et al., 2005).

### Biochemistry and Cellular Function

The ICD of SorLA is important for retrograde trafficking from endosomes to the trans-Golgi network (TGN) by binding to the retromer complex and anterograde trafficking by interacting with clathrin-adaptors (Jacobsen et al., 2002; Seaman, 2007; Fjorback et al., 2012). SorLA binds APP and Aβ to control their transport from endosomes either to the TGN to prevent proteolytic APP-breakdown or to lysosomes for Aβ-degradation, which recently has been reviewed in detail by Schmidt et al. (2016). The mosaic receptor has different extracellular binding domains: an N-terminal Vps10p domain followed by an EGF-precursor homology domain and 11 LBRs. Whereas the LBRs are important for APP binding and rerouting away from the proteolytic pathway (Andersen et al., 2005), the Vps10p domain is responsible for Aβ-binding and the final lysosomal degradation (Caglayan et al., 2014). The Vps10p domain consists of a ten-bladed β-propeller fold with a large tunnel that has a propensity for ligands with a β-sheet formation. An internal ligand derived from the SorLA propeptide binds in this tunnel, extends the domain by one β-propeller blade, and presumably blocks ligand binding (Kitago et al., 2015). The SorLA propeptide is removed in late Golgi compartments by furin (Munck Petersen et al., 1999). SorLA and its interaction with APP have recently been reviewed in detail by Schmidt et al. (2016).

## Very Distinct and Short Receptors Containing LBRs

Lrp3, Lrp10 (murine Lrp9) and Lrp12 (ST7/Mig13) share high homology (Battle et al., 2003) and have two ligand-binding CUB domains, Lrad3 does not have CUB domains (Figure 1). Even though in the literature all four receptors have been claimed to be members of the LDL receptor family, the domain composition puts them into a different class of mosaic proteins. All four receptors lack EGF-precursor homology domains found in all other members of the LDL receptor family. All four receptors have three to five LBRs (Figure 1), but lipoprotein binding remains to be confirmed, and their CTDs encode intracellular sorting motifs. Lrad3 and Lrp10 have been shown to interact with APP, thus we briefly review them in this section.

**Lrp3**, discovered in 1998 is expressed in a wide range of human tissues, including the brain, with the highest expression in skeletal muscle and ovary. Interestingly, in contrast to other LDL receptor family members, Lrp3 does not seem to bind to RAP (Ishii et al., 1998).

**Lrp10 (murine Lrp9)** is expressed in various tissues, including the brain. Little is known about its function; only one publication describes its involvement in APP processing. Lrp10 is located in endosomes and in the TGN (Sugiyama et al., 2000). The cytoplasmic tail interacts with clathrin adaptors that coordinate shuttling between endosomes and TGN (Boucher et al., 2008; Doray et al., 2008). Recently, *in vitro* data showed that APP interacts with the ECD of Lrp10, and both proteins colocalize at the TGN. Lrp10 expression in brains of AD patients is reduced. In cell culture, Lrp10 overexpression induces the accumulation of APP in the TGN, which results in reduced APP-surface expression and processing. Conversely, knockdown of Lrp10 led to increased processing of APP to Aβ (Brodeur et al., 2012).

**Lrp12 (ST7/MG13)** has been annotated as a member of the LDL receptor family in 2003 (Battle et al., 2003). The Lrp12s ICD contains several motifs implicated in endocytosis and signal transduction. Lrp12 is important during CNS development where it controls the formation of the cortical plate, neuronal polarity, and migration (Schneider et al., 2011; Wang et al., 2013b). It is also involved in tumorigenesis including epilepsy-associated gangliogliomas (Garnis et al., 2004; Robens et al., 2016). Silencing of Lrp12 in primary neurons leads to increased dendritic branching, silencing of Lrp12 in the mouse brain during brain development leads to cortical dyslamination and seizure sensitization (Grote et al., 2016). As of today, no role in AD has been described. However, Lrp12 is expressed in neurons and astrocytes of the adult brain (Grote et al., 2016).

**Lrad3** has the shortest ECD of all receptors (Figure 1), with only three LBRs. Lrad3 is found in the brain and is expressed in microvascular endothelial cells and neurons (Otsuki et al., 2005; Ranganathan et al., 2011). In cell culture, the results of Lrad3 overexpression were similar to those of Lrp1: Lrad3 promoted the pathogenic proteolytic pathway of APP, shifting it away from the α-secretase pathway towards the endosome, resulting in enhanced Aβ production. While Lrad3 does not interact with Aβ, the receptor does interact with the central APP fragment (C99) that contains the ICD, the TM-domain, and a short ECD (Ranganathan et al., 2011). The Lrad3-ICD contains two PPxY motifs to which WW-domain containing proteins, e.g., ubiquitin ligases, bind (Ingham et al., 2004). More recently, it was found that Lrad3 is a component of the ubiquitin proteasome system by activating the E3 ubiquitin ligases Itch and Nedd4 (Noyes et al., 2016). However, a direct role of Lrad3 regulation of ubiquitination to APP processing has not been established.

## Lipoprotein Receptors and APP Beyond Alzheimer’S

The function of APP and Aβ beyond AD is not well understood and understudied, especially in conjunction with lipoprotein receptors. Different chapters of this series discuss the physiological role of APP and its cleavage products from various physiological perspectives. APP and its trafficking and processing plays a role in neurite outgrowth and synaptogenesis, APP-deficiency decreases dendritic spine numbers and impairs LTP, which can be rescued by sAPPα but not sAPPβ (Tyan et al., 2012). APP function is largely occluded in single APP mutants, since its paralogs APLP1 and APLP2 can partially compensate for APP-loss. Characterization of combined knockouts of APP and its close relatives APLP1 and APLP2 provides additional insights into the trophic functions of APP: whereas single knockouts and APLP1/APP double knockouts are viable and fertile, combined APLP2/APP or APLP1/APLP2 knockouts display reduced viability (Heber et al., 2000). This suggests that APLP2 carries the most essential physiological functions that can be partially compensated by redundancy in the other family members. APP and APLP2 are expressed ubiquitously, while APLP1 expression is restricted to the nervous system (Lorent et al., 1995). Lrp4, MuSK, Agrin and APP/APLP2 are essential components of a functional complex that recruits and clusters acetylcholine receptors at the NMJ (reviewed in the “Lrp4” Section). Additionally, Lrp4 does not require the KPI domain to bind APP (Choi et al., 2013).

APP trafficking and processing is controlled by a large variety of proteins, but little is known about their physiological relevance. APP interacts with numerous type-I TM receptors, many of which are lipoprotein receptors, and several other ligands, adaptor and scaffolding proteins, which together provide a protein-protein network involved in signaling, processing of various receptors, partially through endocytic pathways.

## Concluding Remarks

APP processing to Aβ and in particular the accumulation of the amyloidogenic Aβ_42_ product, either from increased production or impaired clearance, are initiating events in AD, and ApoE genotype is the most important late onset risk factor for AD. Both APP and ApoE interact with LDL receptor family members to regulate APP trafficking, processing and elimination. Therefore, it is all but certain, that LDL receptor family members play a pivotal role in the pathogenesis of AD.

As a result of the work reviewed in this article, we have learned much about the potential molecular mechanisms that these lipoprotein receptors play in AD pathogenesis, yet the relative importance of each individual event is still unclear. Continuing work on the biology of LDL receptor related genes and their ligands on the physiology of the APP processing machinery holds great promise not only to greater understanding of the disease process but also for the identification of novel and effective therapeutic approaches.

## Author Contributions

TP and CRW jointly wrote the article and designed the figures under JH guidance and JH edited the manuscript.

## Conflict of Interest Statement

The authors declare that the research was conducted in the absence of any commercial or financial relationships that could be construed as a potential conflict of interest.
